# Challenges of promoting physical activity among school children in urban Bangladesh: A qualitative inquiry

**DOI:** 10.1371/journal.pone.0230321

**Published:** 2020-03-16

**Authors:** A. M. Rumayan Hasan, Md. Harunor Rashid, George Smith, Mohammad Abdus Selim, Sabrina Rasheed

**Affiliations:** 1 Health Systems and Population Studies Division(HSPSD), icddr,b, Universal Health Coverage, Mohakhali, Dhaka, Bangladesh; 2 Fragments Magazine, Dhaka, Bangladesh; National University of Ireland Galway, IRELAND

## Abstract

**Introduction:**

Physical activity (PA) confers a multitude of health benefits. Unfortunately, Bangladeshi school children get little PA. The current study assessed the barriers to promoting PA from the perspectives of school authorities and parents in urban Bangladesh.

**Materials and methods:**

This study was conducted between January-June 2018 in Dhaka city. Fourteen schools were sampled to represent different school types. We used qualitative methods: 14 key Informant interviews (teachers), six focus group discussions (parents), and 14 observations of school environments. Thematic analysis was performed.

**Results:**

PA was not prioritized at most schools for two primary reasons: 1) there was a general lack of understanding of the importance of PA; and 2) physical education classes did not contribute to grades. Little time and resources were allotted for physical education classes because little priority was given to PA by school authorities. Outside school, lack of adequate safe open spaces undermined access to PA. Further, there were social barriers to PA including lack of parental support due to concerns about tiredness and injury and the perception that PA was detrimental to academic achievement. Many parents chose screen-based activities for children over PA.

**Conclusion:**

Despite policies that mandate PA, PA lost out to school and parental priorities for academic achievement. Parental concerns about tiredness, injury and safe spaces impeded children’s access to PA outside of school. Steps should be taken to enforce existing policies that mandate effective PA for school children, and parents and teachers should be educated about the need for and benefits of PA.

## Introduction

Physical activity (PA) confers a multitude of health benefits to young children and adolescents [1, 2]. Regular PA improves cardiovascular health, mental health and muscular strength and flexibility [3, 4]. PA calorie expenditure helps control weight [3]; better weight control could contribute to reducing overweight and obesity. PA can also help children and adolescents develop social skills, teamwork and leadership [5]. The World Health Organization (WHO) recommends that children and adolescents should have at least 60 minutes of moderate-to-vigorous PA, daily [6, 7]. Most children and adolescents around the world fail to meet the WHO recommendation [8] and in Bangladesh only 41% of young people meet it [9, 10]. Promoting PA among the young can have lifelong benefits as healthy habits formed early in life can continue into adulthood [11].

Researchers have shown that participation in PA by children and adolescents depend both on social and environmental factors and on individual factors such as age, sex, ethnicity, immigration status, parental income and education [12, 13]. In recent years growing evidence has associated the physical environment with levels of PA among children. Some researchers have reported that proximity and accessibility to recreational facilities, traffic volume, residential density and neighborhood walkability affect children’s PA [14]. Researchers have also examined the association between different school characteristics such as availability of equipment, space, school size, number of teachers and programs and policies related to PA. Findings have been inconsistent, however [15]. To our knowledge, no study in Bangladesh has looked at factors associated with PA both in schools and neighborhoods in order to understand the pathways that affect children’s PA. The influence of social factors on PA has been described by researchers around the world. A review of literature from Arab countries and among the Indian diaspora living in Canada showed the importance of gender roles, religious identity and cultural norms shaping support for PA for children [16, 17]. Researchers have reported that parental support for PA is positively associated with PA among children [12, 18]. This support includes encouragement, providing money and transportation, watching children perform PA, and actively participating in PA with children [19]. Researchers have reported that parents positively affect children’s PA by modeling PA themselves [20]. Friends and peers can also positively or negatively impact children’s PA [21]. However, information about social factors that affect PA among Bangladeshi school children is scarce.

Several studies from Bangladesh have explored risk factors for rising overweight and obesity among school children. Researchers have reported that rapid urbanization, decreasing number of playgrounds, increasing purchasing power and therefore, easy access to mobile phones and hand-held computers are potentially important contributors to low PA among Bangladeshi children [22, 23]. Though PA is a mandatory part of the education curriculum in Bangladesh and schools are required to employ trained teachers to provide physical education classes [24], little is known about implementation of the policy. Scant information is available from Bangladesh about environmental and social barriers to PA for school children. Given the importance of both social and environmental factors in predicting young children’s and adolescents’ PA participation, our current study was designed to explore the challenges of promoting PA among school children from the perspectives of school authorities and parents in urban Bangladesh. The insights from the study will help to inform policies and programs for promoting PA among children and adolescents in Bangladesh and other low resource settings.

## Materials and methods

### Study design and study site

The current study was part of a mixed method, cross sectional study designed to assess the risk factors of overweight and obesity among school children, with a focus on PA and healthy food choices. For our current analysis we looked only at qualitative data related to PA. The study was conducted from January-June 2018. Dhaka city administratively divides into two areas: North and South. We purposively selected one thana (area under one police station) from each administrative area based on availability of different types of schools. For our study, we selected Uttara thana and Ramna thana from Dhaka North and Dhaka South, respectively.

### Participants and procedure

We mapped all schools in the study area and stratified them by school fees into high-tuition, medium-tuition and low-tuition. Our assumption was that school fees would represent both the resources available to the schools and the socioeconomic status of the families the students came from. During random selection, if a girl’s school was chosen, an additional boy’s school of the same stratification was chosen from the same area. Fourteen schools were selected for the study (Table 1).

**Table 1:** Sampling of schools by study area medium of instruction, types of school and tuition.

Characteristics	# of High-tuition schools	# of Medium-tuition schools	# of Low-tuition schools
Areas			
Dhaka North	2	2	3
Dhaka South	2	2	3
Medium of instruction			
English only	3	0	0
Bengali/English	1	0	0
Bengali only	0	4	6
Types of school			
Co-education	4	4	4
Single sex	0	0	2

We contacted authorities from all selected schools and provided formal letters describing the study objectives and invited them to take part. Among schools willing to participate in the study we conducted key informant interviews (KIIs) with school teachers and principals, focus group discussions (FGDs) with mothers and, checklist-based structured observation of the PA facilities on school premises. The checklist covered information about the PA activities that occurred at school, content of physical education classes, the availability of a trained PA teacher, the playground and the encouragement given female students. The checklist was developed based on literature review and field tested to ensure its applicability in our context. To recruit study participants we initially approached the principal of each school to recruit him/her as a key informant because he/she was responsible for the overall administration of the school. After informing the principals about the requirements of the study, they recommended that we recruit physical education teachers. We explored the principals’ and teachers’ concerns about the promotion of PA in school and the challenges they face for such promotion. Although we intended to have both fathers and mothers in the FGDs, only mothers were available on school premises (as they waited for their children). Therefore, only mothers were FGD participants. We used purposive sampling to recruit the participants for both KIIs and FGDs.

### Data collection

For qualitative data, KIIs were conducted face-to-face in a place convenient for the respondent (usually his/her office); FGDs with mothers were held in parent waiting areas adjacent to the schools; and structured observations were conducted at all schools selected for the study (Table 2).

**Table 2:** Qualitative methods and sample sizes.

Methods	Foci		Type of school	
		High-tuition school	Medium tuition school	Low tuition school
KII (key informant)	Principal	3	3	2
	Phys Ed teacher	2	2	2
FGD	Mothers (6–12 mothers/FGD)	2	2	2
Observation	PA facilities in school	6	4	4

The qualitative field team consisted of 4 people (2 men, 2 women) who hold Masters in Anthropology or social science and had 4–5 years experience collecting qualitative data; they were trained by an anthropologist (RH) and a public health nutritionist (SR). KIIs and FGDs were conducted using separate open-ended guidelines developed based on literature review and experience from previous studies. The KII guideline was designed to explore the following topics: PA opportunity within school, physical education classes, attitude towards PA and barriers and facilitators to promoting PA within school. The FGD guideline was designed to explore the following topics: parental understating of and attitude towards PA, and perceived barriers and facilitators to promoting PA both within and outside of school. The guidelines both for KIIs and FGDs, were pre-tested in a school (not part of the study sample) for finalization. During iterative analysis, issues that had not been considered initially but emerged during data collection and preliminary data analysis were incorporated into the guidelines both for KIIs and FGDs. Extensive field notes were taken during the interviews and FGDs. The KIIs took 40–50 minutes to conduct and the FGDs 60–90 minutes.

### Data analysis

**Fig 1:**
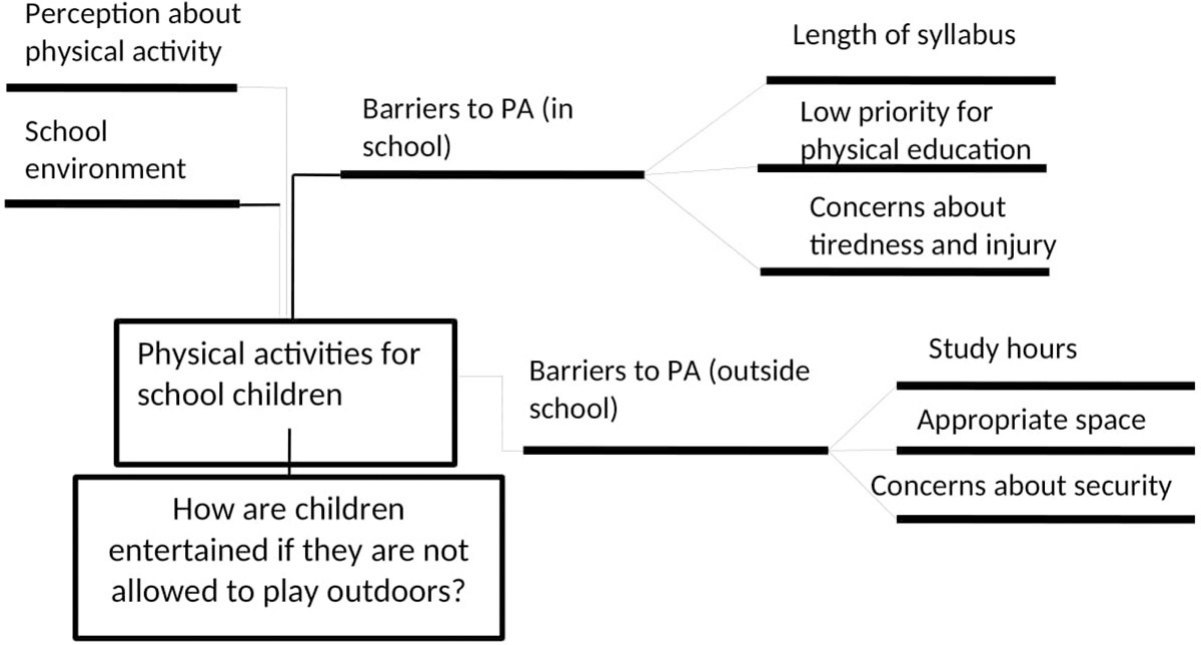
Themes of qualitative inquiry.

All interviews were electronically recorded and transcribed in Bengali verbatim. The data were analyzed using inductive thematic analysis procedures manually [25]. We chose this analytic procedure as it produces a rich description of data and themes. We followed the following steps for thematic analysis [26]: a) familiarized ourselves with textual data through repeated readings; b) generated initial codes and collated data under each code; c) sorted all coded data and extracted the data into themes and sub themes related to social and environmental barriers to PA; d) reviewed themes and formatted a thematic matrix for further analysis; e) labeled themes. Triangulation was achieved by comparing and contrasting findings from different methods. The transcribed data were checked for accuracy by two investigators (RH and AS) who listened to the recordings while reading the transcripts. All transcripts were coded independently by two investigators (RH and AS) under the supervision of a topic expert (SR). The investigators cross checked each other’s coded transcripts and met as a group to resolve any discrepancies in coding. Regular peer debriefing was conducted within the study team to help understand the issues and to interpret the findings. The final themes and sub-themes are provided in Fig 1.

### Ethics statement

All participants provided informed written consent before data collection commenced. All participants were given time to read the consent form and to ask questions. Then they were asked to sign the consent form. In this study we did not collect any other identifiable information from the participants. In order to maintain participants’ anonymity, we allocated an identification number to each participant. Collected data were kept in a secure location and were only accessible to the research team. The study received ethical clearance from the Ethical Review Committee of icddr,b.

## Results

All FGD participants were mothers. Most were 30–39 years of age, were stay-at-home mothers, and had 11–15 years of education. The key informants were teachers who were mostly men, were older than 40 years, and had more than 11 years of education (Table 3).

**Table 3:** Profile of respondents.

Characteristics		
	Focus group discussion (n = 31)	Key informant interview (n = 14)
***Age group***		
20–29	8	0
30–39	16	0
40–49	7	10
50–59	0	4
***Gender***		
Male	0	9
Female	31	5
***Profession***		
Stay-at-home mother	24	0
Service	7	0
Teacher	0	14
***Years of schooling***		
6–10	11	0
11–15	20	14

### Physical activities for school children

#### Perceptions about physical activity

Most mothers believed that PA was needed for the health and wellbeing of their children. However, very few mothers could explain how PA actually affected the health of their children. When discussing types of PAs that interested their children, mothers mentioned running around the field, playing any outdoor or indoor games, participating in school activities and moving around the house. A few mothers also mentioned local indoor sedentary activities such as *Ludu or caram*. They did not mention walking to school as a physical activity. Only one mother talked about making special effort to ensure that PA was a regular part of her child’s life:

*I enrolled my child in the cricket club in our area*. *I bought him cricket gear*. *I take him to play regularly*. *In order to stay fit and healthy*, *it is important (to play)*. (Mother, medium-tuition school, FGD-3)

When we asked the teachers about PA among children they talked about games, transportation, participation in physical education, participation in parades and running within the school.

#### School environment

About half the schools in the sample did not have their own playgrounds (Table 4). Among the schools that had playgrounds on their own premises, only the high-tuition schools held regular, organized activities, while medium- and low-tuition schools held annual sports events after final examinations in December and January. Despite not having their own playgrounds, all the school authorities reported having physical education classes for grades 6–10 and employed teachers for those classes. However, we found that only high-tuition schools held organized PAs (such as exercise or games under the supervision of a teacher) in physical education classes. Only two schools held sports clubs for their students on school premises during weekends. Further, few schools stated that they encouraged female students to participate in organized PAs.

**Table 4:** Observation of facilities for physical activities in school.

Facilities available	High tuition schools (n = 4)	Medium tuition schools (n = 4)	Low tuition schools (n = 6)
Play ground within school premises	3	2	3
Organized activities			
*Regular*	3	-	-
*Occasional*	-	2	3
Scheduled physical education classes	4	4	6
Physical education teacher employed	4	4	5
Organized activities in physical education classes	3	0	0
Female participation encouraged	2	1	0

The schools that did not provide facilities for PAs, but had at least a bit of open space, claimed that they offered exercise for students at the beginning of the day during assembly. A teacher from a medium-tuition school stated:

*We do not have opportunity to involve children in physical activity*. *Therefore*, *we added some exercise into the assembly*. *Now our students get some physical activity at the school*. (Principal, medium-tuition school, KII-4)

In schools where no open space was available for play, the lack of space was a barrier to PA. In these schools, assembly at the beginning of the day was held in classrooms, which precluded exercise. As one teacher from a low-tuition school said:

*We do not have required environment for physical activity. Our students only get time during tiffin breaks (recess). They cannot even stand properly (due to space limitation). Our school is in a single building*. (Principal, low-tuition school, KII-2)

#### Barriers to PA (in school)

*Length of syllabus*. Teachers struggled to finish large syllabi within the school year. Scheduled examinations took up much of the school calendar and teachers’ time. To finish their syllabi on schedule, school authorities scheduled classes continuously, often taking time away from physical education classes. As one teacher explained:

*We do not have any scope of playing (during school hours). The time the students spend (at school) is used for studying only. We have very little time and the government has set a schedule of six classes (per day). So (we) cannot give a break. How can students spend time playing?* (Principal, medium-tuition school, KII-3)

A few teachers said that students had limited opportunity for PA during recess because recess was used for lunch. One teacher from a private school added that the compact class schedule with little time for breaks was a result of having two shifts of school where the morning shift ran from 7:30am-12pm and afternoon shift ran from 12pm-4pm. According to this teacher running two shifts hampered teachers’ ability to provide either physical education or adequate breaks.

*Low priority for physical education*. All the schools had physical education and health as integral parts of the syllabus for grades 6–10. Teachers were hired to offer 2–3 physical education classes per week for each grade. However, the lack of space meant that teachers would only impart the theoretical parts of the syllabi and not the games or exercise. A few of the teachers said that since physical education did not contribute to final grades it received low priority. As one principal from a low-tuition school complained:

*You will find that physical education and health class is in the routine and teachers are available but the class does not take place. The guardians do not want this class. They (guardians) think that sports class kills crucial time (that can be spent studying)… Children should be focusing on activities that contribute to good grades. As parents perceive that physical education and health class does not impact on grades they put pressure on us to remove it (from the schedules). We are forced (to remove the class).* (Principal, low-tuition school, KII-10)

So for all groups with decision making power–guardians and school authorities–physical education was not deemed a priority.

*Concerns about tiredness and injury*. From mothers and teachers we heard that parents’ perception of tiredness and injury was a barrier to PA. Mothers complained that sports increase tiredness and reduce concentration during class, so they discouraged their children from participating in PA. According to one principal:

*Parents and teachers think that a student should spend all his time for academics. It is true that children would get tired (after games) but parents believe that tiredness is a barrier (to study). Guardians put pressure on us (to remove those activities). They do not allow their children to take part (in PA).* (Principal, low-tuition school, KII-10)

Some of the guardians feared injuries during games. This sometimes led to changes in the activities offered in physical education curricula. A mother and physical education teacher commented:

I don’t let him play because they get injured and have accidents. That’s why I am not interested in the games. Children don’t want to participate (in games at school) as they know that if they get hurt, I will scold them. *(Mother*, *high-tuition school*, *FGD-1)*

*Earlier we offered long jump and high jump every winter for high school students. In the previous season we removed those games. Guardian(s) complained that there is (the) possibility of injury. One student got (a) light injury while running (a 100-meter race). So we reduced the length of sprint from 100 to 80 meters. We want to create space of physical activities for our students but it is not possible in the current environment*. (Physical education teacher, low-tuition school, KII-11)

A few mothers also mentioned that they did not like the fact that their children got dirty during games and so they did not allow them to participate in school sports.

#### Barriers to PA (outside school)

*Study hours*. The priority given to academics led to additional time studying outside of school. Most students spent substantial time after school attending coaching centers or studying with private tutors. The tight schedules reduced opportunities for PA after school. As a mother explained:

*After school, my son comes home, showers and eats lunch. He studies until 6pm. Then he takes a prayer break and sometimes eats a snack. Soon afterwards his house tutor comes. When does he have time (to play)?* (Mother, medium-tuition school, FGD-3)

A few mothers also talked about long school hours and the commute home making children tired and less interested in PA.

*Appropriate space*. A few mothers brought up the lack of open space in their locality as a barrier to PA. One mother said:

*Where will he play …? We do not have (a) playground (in the area). He has time in the afternoon, but he cannot use it to play outside.* (Mother, low-tuition school, FGD-6)

*Concerns about security*. In the localities where open spaces for playing were available, mothers expressed concern about security. Mothers feared kidnapping and valuables being stolen from their children while they played. As a result parents refused to send their children to play in local fields when either they themselves were too busy or reliable caregivers could not chaperone. In the words of one mother:

*I cannot let my child go alone to play (in the field)*. *There may be kidnappers there*. *If I want him to play*, *I have to go (with him)*. *I have a lot of work in the afternoon*, *so it is a problem to send him (to the field)*. (Mother, high-tuition school, FGD-4)

A few mothers also expressed concerns about children picking up bad habits such as smoking and drugs from people they might befriend at the local fields. These safety concerns were mostly expressed by mothers of children from high- and middle-tuition schools. Children who played in the streets and alleys were mostly from low socio-economic status households. One teacher linked social class with use of available spaces for PA:

*Children from low social class play in the street. My son often watches children his age playing in the alley from the balcony, but he does not join (them)…I do not allow him to play on the street as there are cars and rickshaws…They (kids from the lower class) are used to playing (on the street) so they play. I can’t allow him*. (Physical education teacher, low-tuition school, KII-11)

How are children entertained if they are not allowed to play outdoors?

Many parents allowed their children to watch television, or play games on computers or mobile phones. The screens entertained children while keeping them safe at home. As one mother with a child in a medium tuition school said:

*We are helpless…What will we do? We do not let our children go (to the field)…We worry about the environment and that our children may be harmed. If they stay home we feel relieved…At least they are in front of our eyes. They watch cartoons or play games (on a computer).* (Mother, medium-tuition school, FGD-2)

Similar sentiments were expressed by a teacher who spoke specifically about how this perceived lack of security adversely affected children’s access to PA.

*Although there are fields, many bad things happen there. Parents do not feel comfortable about taking children to play there …They (fields) are not safe. What if children acquire bad company and drug habits by going to play (in the field)? So parents keep the kids home and make them dependent on electronic devices…So they don’t get to play or move around*. (Physical education teacher, high-tuition school, KII-13)

Some mothers also spoke about using electronic devices to entertain children or to feed picky eaters:

*Most of the children are used to eating while watching cartoons on TV or other devices* …*This is the easiest way to feed children*. (Mother, high-tuition school, FGD-4)

A teacher observed that parents had little time for their children and provided the different devices to compensate for the lack of parental attention:

*Parents give the smart phone to children to keep them busy…They (parents) are busy with work and do not have time (to spend with their children). Children get used to gaming and watching TV. Soon they are not interested in playing outside games and parents are happy that their children spend time at home*. (Physical education teacher, medium-tuition school, KII-5)

The extensive access to electronic devices at home also resulted in reduced interest in PA among children even when it was offered. As a teacher from a high-tuition school said:

*I observed that some students stay in the class room (during break). We cannot force them to take part in the physical activity. I think those students are accustomed to sedentary activity (using electronic devices) at home. They lost interest in playing (in the field)’.* (Principal, high-tuition school, KII-7)

#### Programmatic implications

Based on our findings, we believe efforts are needed to make PA a priority for both school authorities and parents (Table 5). It is important to hold PA classes regularly and to keep space constraints in mind when constructing PA curricula. Further, teachers should be trained to find opportunities for PA no matter how limited the space and trained to keep these activities as safe as possible to reduce the risk of injury and assuage parents’ concerns. There also needs to be an articulation of the fact that the risk of sedentary life outweighs the risk of injury from PA for both long and short term health and wellbeing. Finally, community open spaces should be made available and safe for children, with specific attention to the safety issues, actual and perceived, endemic to the society.

**Table 5:** Programmatic implications of the findings.

Areas of interest	Constraints/ barriers	Favorable factors	Opportunities for intervention
Perceptions	• Mothers did not understand the link between physical activity and school performance • Mothers did not prioritize physical activity	• Mothers linked physical activity with health • Mothers prized academic achievement	• Behavior change communication materials and targeted activities should be designed to link physical activity and school achievement for parents
School environment	• Lack of appropriate space	• Physical education is part of the curriculum • Phys Ed teachers are employed	• Create modules for PA taking space constraints into account • Train Phys Ed teachers to deliver the modules
PA at school	• Low priority • Parental concern about tiredness and injuries • Tight class schedule limits time	• Teachers were aware of the need for physical education	• Include assessment criteria for PA in final class assessments • Outreach to parents and school boards • Link PA to academic achievement
Participating in PA/sports outside school	• Lack of open space • Security concerns • Priority given to academic activities • Screen-based activities occupy children	• Parents are concerned about the health of their children • Children are interested in playing	• Safe play areas for children should be identified and promoted • School fields should be made available after school hours for the community • Different types of PA should be made available at community open spaces • Raise awareness in the community about the detrimental effect of prolonged screen watching • Engage community leaders to promote PA

## Discussion

According to our study’s findings, school children faced environmental and social barriers which affected their participation in PA both in and outside schools. These barriers need to be addressed to increase children’s PA and, thereby, both stem the rising tide of overweight and obesity among school children in Bangladesh and contribute to overall health throughout the population [27, 28]. In previous studies addressing urban Bangladeshi school children, researchers reported a high prevalence of overweight and obesity [29, 30] and identified sedentary activities as a risk factor [23]. Researchers also reported that regular PA can protect against overweight and obesity [31, 32]. To our knowledge, ours is the first study to explore the environmental and social barriers that affect PA among young school children and adolescents in Bangladesh.

Although physical education class is a mandatory part of school curricula, we found the policy largely ignored. Since PA does not contribute to grades it was given low priority and mostly did not take place. The pressure to finish school curricula and the focus on academic achievement undermined physical education classes and reduced PA in schools, a finding similar to United States’ study where school authorities’ competing priorities were barriers to PA at schools and adequate PA lost out to academic work [33]. In our study, some schools with adequate space failed to use it regularly for PA as the structure of the school day did not allow breaks of sufficient length for students to play. Researchers from other Asian countries have also reported that quality physical education was lacking. The barriers for implementing quality PA included physical education not being a mandatory subject in school, teachers’ lack of understanding of the importance of physical education, lack of funding, time allocation, and staffing, inadequate facilities, low salaries and low social recognition for sports teachers [34–36]. For schools with inadequate space for play, PA on school premises is difficult. Similar findings about lack of space and equipment being barriers to PAs at schools have been reported by researchers in developed countries [15, 37]. The education policy in Bangladesh already requires schools to have mandatory physical education classes, employ physical education teachers, provide fields for games (for new schools) and allocate budget for physical education classes [24]. However, the policy is poorly enforced. For effective enforcement of the policy there must be political commitment to the importance of PA. It will be important to monitor, among other indicators, the actual amount of PA that school children participate in. Further, it is important to find ways to link the accountability mechanism for effective PA to school funding and/or licensing to push schools to adequately enforce the policy.

Outside of schools, parents expressed concern about the safety of community open spaces. They were concerned that their children would be exposed to crime and drugs and worried about abduction if children went to play without supervision. Similar concerns about the safety of open community spaces have been reported by researchers in other countries [38, 39]. With nuclear families becoming more prevalent and increases in the number of working women [40, 41], providing adequate supervision for children has become difficult for urban parents. It is vital to assess the safety of open community spaces, to take measures to increase their safety, and, if there is a gap between actual and perceived safety, endeavor to close that gap. Promoting organized PA under trained coaches in open community spaces can make spaces safer and also increase the perception of safety, as people see more activities taking place and that those activities are organized and responsibly supervised.

Beyond the identified environmental barriers, our study indicated that there were important social barriers that affected PA participation among school children and adolescents. Parents in our study expressed concerns about PA leading to tiredness and injuries, but these concerns need to be considered in the context of the overwhelming cultural emphasis on academic achievement. From mothers’ perspectives, a tired child cannot concentrate on studies so PA becomes a barrier to academic achievement. The negative perception of the link between academic achievement and PA leads to parental pressure on schools to reduce PAs and reduces parental support for after school PA. In Nepal researchers have reported similar findings about parents prioritizing homework and their concerns about injury leading to inadequate emphasis on PA [42]. Others have reported that among Middle Eastern and Chinese immigrants to Australia, parental focus on academic achievement meant that they did not permit or encourage their children to participate in PA [43]. A review of interventions to promote PA among children and adolescents revealed that an increasing number of school-based interventions to promote PA included some parental involvement [44] as a response to the strong influence of parents on children’s activities. However, usually these interventions were limited to sending parents informational materials and homework assignments [44]. It is likely that for these interventions to be more effective, parents and school-teachers need to be more actively engaged and educated. Cultural norms related to girls’ participation in PA in general, and as they reach pubescence in particular were not explicitly discussed by our respondents but should be addressed in the future as researchers have reported religious norms shaping girls’ access to PA in other Muslim majority societies [17]. And it is imperative that effective interventions be found as, counter to many parents’ and educators’ beliefs, PA has the potential to improve academic achievement and cognitive development [45, 46] and to reduce the risk of children being overweight and obese [32].

Over the last few decades, Bangladesh has made great strides in enrollment and retention of children at primary and secondary levels [47]. In grades 1–5, the enrollment rate increased from 87% in 2005 to 98% in 2016 and the dropout rate decreased from 47% in 2005 to 19% in 2016. Similarly, in grades 6–10, the enrollment increased from 45% in 2008 to 68% in 2016 and dropout rate decreased from 61% in 2008 to 38% in 2016 [47]. With these high enrollment rates, schools are an excellent venue for reaching children in Bangladesh with messages about and practices for a healthy lifestyle. Strong PA curricula in schools may help inculcate lifelong habits of PA and reduce the risk of future diseases and disabilities in the Bangladeshi population. In view of the rise in overweight and obesity among children in urban areas [29, 30] and the rise in deaths due to non-communicable diseases in Bangladesh [48], it is important that strong steps be taken to improve the opportunities for PA for school-aged children.

The strength of our study is that the barriers to PA among school children have been assessed from the perspectives of parents and teachers who can strongly influence children’s PA participation. The use of qualitative methods allowed us to understand the nuances of the pathways of influence of various factors linked to PA. There were some limitations to our study. First, we were unable to reach fathers as they were not available during school hours and, therefore, we missed their perspectives on PA. Second, we did not explore the children’s perspective of PA which would have given us insights about the barriers to PA from their perspective. Finally, we did not include the perspectives of opinion makers such as local social and religious leaders in our study.

## Conclusions

Although physical education is a mandatory part of school curricula, it was prioritized neither by schools nor mothers. This lack of prioritization has resulted in little time and few resources allocated for PA. It is important that the policy for physical education classes be enforced to ensure adequate PA for school children. Outside of school, community open spaces were inadequate and considered unsafe by parents, which is another important barrier to PA for children. We found parental concerns about tiredness and injury related to PA and parents’ lack of understanding of the importance of PA and its link to academic achievement impeded children’s access to PA both in and outside school. The solution to the larger problem of promoting PA in the population of Bangladesh and in other low resource settings may lie in creative engagement with stakeholders from multiple sectors such as education, city planning, the private sector, health and law enforcement, parents and children, themselves.

## Supporting information

S1 Data Guidelines of FGD and KII. (DOCX)
