# Supplementary material for: Challenges of promoting physical activity among school children in urban Bangladesh: A qualitative inquiry
Source: PLoS One. 2020 Mar 16;15(3):e0230321. doi: 10.1371/journal.pone.0230321 (PMC7075558; doi:10.1371/journal.pone.0230321)
Supplement: S1 Data — (DOCX) [file pone.0230321.s001.docx]

**Guideline for FGD**

1. What do you think about the physical activity of your children? What is your opinion about your child’s physical activity every day?
2. What is the role of physical activity or active play in a child’s development? How important do you think it is for children?
3. What sort of facilities for PA is available in schools for children? (space, organized/unorganized, regular/seasonal)
4. What types of physical activities are children engaged in school? What are the challenges? How can they be overcome such?
5. How do your children spend time at home? Outside school what types of physical activities do your children engage in every day? (At home/outside home)? What are the barriers related to PA outside school? How can one overcome such barriers?

**Guideline for FGD (Bengali)**

1. Avcbv‡`i wkï‡`i kvixwiK PP©v m¤ú‡K© Avcbviv wK g‡b K‡ib? wkï‡`i cÖwZw`‡bi kvixwiK PP©v m¤ú‡K© Avcbv‡`i gZvgZ wK?
2. wkï‡`i weKv‡ki †ÿ‡Î kvixwiK PP©v ev †Ljva~jvi f~wgKv KZUzKz Av‡Q? wkï‡`i Rb¨ G¸‡jvi ¸iæZ¡KZUzKz?
3. wkï‡`i Rb¨ ¯‹z‡j kvixwiK PP©vi wK wK my‡hvM myweav Av‡Q? (‡Ljvi RvqMv, msMwVZ/AmsMwVZ, wbqwgZ/‡gŠmg wfwËK †Ljva~jv)
4. ¯‹z‡j †Kvb †Kvb †Ljvq wkïiv AskMÖnY K‡i, AskMÖn‡Yi †ÿ‡Î wK wK cÖwZeÜKZv ev evav Av‡Q e‡j g‡b K‡ib? ¯‹z‡j kvixwiK PP©v Kivi ‡ÿ‡Î evav/ cÖwZeÜKZv¸‡jv wKfv‡e KvwU‡q IVv m¤¢e?
5. Avcbv‡`i wkïiv evmvq wKfv‡e mgq KvUvq? Avcbv‡`i wkïiv ¯‹z‡ji evB‡i cÖwZw`b wK ai‡Yi kvixwiK PP©v K‡i _v‡K? (evmvq/evmvi evB‡i?) ¯‹z‡ji evwn‡i kvixwiK PP©vq AskMÖn‡Yi †ÿ‡Î wK wK evav ev cÖwZeÜKZv Av‡Q?

evavmg~n wKfv‡e `~i Kiv hvq?

**Guideline for Key Informant Interview (KII)**

1. What do you think about school children’s PA?
2. What facilities for PA are available in your school?
3. What are the challenges that impede implementing physical activities at school? How can such challenges be overcome?
4. What types of activities are children engaged at your school within physical education classes? Do you think this is sufficient, if yes why? If no, what are the barriers?
5. What kind of policies does your school have about physical education?
6. How can children increase physical activity during school?

**Guideline for Key Informant Interview (KII) (Bengali)**

1. ¯‹zj wkÿv_x©‡`i kvixwiK PP©v m¤ú‡K© Avcwb wK g‡b K‡ib?
2. Avcbvi ¯‹z‡j kvixwiK PP©vi wK wK my‡hvM myweav Av‡Q?
3. ¯‹z‡j kvixwiK PP©v¸‡jv ev¯Íevq‡b evavmg~n wK wK? wKfv‡e GB evav¸‡jv `~i Kiv m¤¢e?
4. kvixwiK wkÿv K¬v‡ki PP©v¸‡jvi g‡a¨ wkÿv_©xiv †Kvb ai‡Yi PP©v¸‡jv K‡i? Avcwb wK Zv h‡_ô e‡j g‡b K‡ib? hw` nu¨v nq †Kb? hw` bv nq, cÖwZeÜKZv¸‡jv wK wK?
5. kvixwiK wkÿv wel‡q Avcbvi ¯‹z‡j wKai‡Yi bxwZgvjv Av‡Q?
6. ¯‹z‡j kvixwiK Kvh©µg wKfv‡e evov‡bv hvq?
